# Modulating gut microbiota and metabolites with dietary fiber oat β-glucan interventions to improve growth performance and intestinal function in weaned rabbits

**DOI:** 10.3389/fmicb.2022.1074036

**Published:** 2022-12-15

**Authors:** Li Ma, Zhengzhong Luo, Yixin Huang, Yan Li, Jing Guan, Tao Zhou, Zhenlong Du, Kang Yong, Xueping Yao, Liuhong Shen, Shumin Yu, Zhijun Zhong, Yanchun Hu, Guangneng Peng, Xiaodong Shi, Suizhong Cao

**Affiliations:** ^1^Department of Clinical Veterinary Medicine, College of Veterinary Medicine, Sichuan Agricultural University, Chengdu, China; ^2^Key Laboratory of Coarse Cereal Processing, Ministry of Agriculture and Rural Affairs, Chengdu University, Chengdu, China; ^3^School of Biodiversity, One Health and Veterinary Medicine, College of Medical, Veterinary and Life Sciences, University of Glasgow, Glasgow, United Kingdom; ^4^Department of Animal Husbandry and Veterinary Medicine, College of Animal Science and Technology, Chongqing Three Gorges Vocational College, Chongqing, China

**Keywords:** oat β-glucan, gut microbiota, metabolites, intestinal function, growth performance

## Abstract

The effect of oat β-glucan on intestinal function and growth performance of weaned rabbits were explored by multi-omics integrative analyses in the present study. New Zealand White rabbits fed oat β-glucan [200 mg/kg body weight (BW)] for 4 weeks, and serum markers, colon histological alterations, colonic microbiome, colonic metabolome, and serum metabolome were measured. The results revealed that oat β-glucan increased BW, average daily gain (ADG), average daily food intake (ADFI), and decreased serum tumor necrosis factor-α (TNF-α) interleukin-1β (IL-1β), and lipopolysaccharide (LPS) contents, but did not affect colonic microstructure. Microbiota community analysis showed oat β-glucan modulated gut microbial composition and structure, increased the abundances of beneficial bacteria *Lactobacillus*, *Prevotellaceae_UCG-001*, *Pediococcus*, *Bacillus*, etc. Oat β-glucan also increased intestinal propionic acid, valeric acid, and butyric acid concentrations, decreased lysine and aromatic amino acid (AAA) derivative contents. Serum metabolite analysis revealed that oat β-glucan altered host carbohydrate, lipid, and amino acid metabolism. These results suggested that oat β-glucan could inhibit systemic inflammation and protect intestinal function by regulating gut microbiota and related metabolites, which further helps to improve growth performance in weaned rabbits.

## 1 Introduction

Young animals, especially weaned animals are vulnerable to pathogens due to the immaturity of the digestive system and immune system, which always causing intestinal mucosal damage and gut microbial dysbiosis, and further leading to growth retardation or even death of animals (Ren et al., 2018). Prebiotics are considered as substrates that are selectively utilized by host microorganisms, in the process host gut microbiota also be restructured, increasing beneficial bacteria abundances but inhibiting the growth of pathogens (Sanders et al., 2019). In addition, consumption of some prebiotics, like dietary fiber, could produce short-chain fatty acids (SCFAs), which can act to improve barrier function in the gut and modulate immune cell activity (van der Hee and Wells, 2021; Johnson et al., 2022). Based on this, prebiotics can be used as non-antimicrobial alternative feed additives in young animal rearing to promote host health.

Beta-glucan is regarded as a dietary fiber that has prebiotic potential, and cereals, especially oat, are the main source of β-glucan (Bai et al., 2021a). The U.S. Food and Drug Administration, the Joint Health Claims Initiative, and the French Food Safety Agency have approved health claims for oat β-glucan as it presents a broad range of biological activities, such as lowering blood cholesterol, anti-diabetes, immunomodulatory, and anti-inflammatory activities (Jayachandran et al., 2018; Yu et al., 2022). Nowadays, accumulating researches have also suggested that it has potential health promoting properties on intestinal function. For example, supplementation with whole oat meal or β-glucan induced increase in intestinal Na^+^ K^+^-ATPase activity, Ca^2+^ Mg^2+^-ATPase activity, and energy charge in rats (Zhang et al., 2012). Several studies have also proposed its role in gut development and microbiota regulation; a previous study suggested that oat β-glucan treatment not only increased duodenum and ileum villus height/crypt depth ratios but also showed higher expression levels of genes associated with intestinal barrier function (*zonula occludens 1* and *claudin 1*) in weaned pigs (Wu et al., 2018). Additionally, dietary oat-derived β-glucan was found to alter the colonic bacterial community of mice, which increasing the phyla Bacteroidetes and Proteobacteria, while decreasing the phyla Firmicutes in BALB/c mice (Luo et al., 2017).

The functionalities of oat β-glucan are inextricably related to gut microbiota and its catabolite. Bai et al. (2021b) found that oat β-glucan ameliorated dextran sulfate sodium (DSS)-induced colitis in mice simultaneously by regulating gut-derived SCFAs and microbial metabolic biomarkers. Another study pointed out that oat β-glucan treatment could modulate intestinal microbiota toward a healthier profile, promote the growth of *Lactobacillus* and the release of butyrate, which could ameliorate inflammation and consequently improve renal function in diabetic nephropathy rats (Wang et al., 2022). However, to our known, only a few studies reported the role of oat β-glucan fermentation in the intestinal function. Shen et al. (2012) found oat β-glucan exerts favorable effects on improving intestinal function and health by promoting intestinal SCFAs production and improving colonic *Bifidobacterium* and *Lactobacillus*, lowering colonic *Enterobacteriaceae* counts, but their study only focused on a few specific bacteria and metabolites due to the limitation of research technology. As intestinal function is highly associated with animal growth (Zou et al., 2022), there is still need a comprehensive study to systemically explore whether oat β-glucan exerts its beneficial effects on intestinal function *via* microbiota and its metabolites, as well as its effect on the overall metabolism of the body.

Rabbit is an animal model that is widely used in clinical trials. It does not secrete cellulolytic enzymes by itself, and mainly depends on bacteria in the hindgut for the utilization and digestion of dietary fiber (Liu et al., 2022). On the other hand, rabbit meat is thought to be a functional food as it rich in highly digestible protein, essential amino acids, unsaturated fatty acid, calcium and phosphorus, while contains less fat, cholesterol and sodium when compared to traditional red meat. These nutrients properties of rabbit meat also meet people’s demand of having a healthy lifestyle, thus promoting the consumption of rabbit meat in the worldwide (Nasr et al., 2017; Krunt et al., 2022). The current study therefore used a weaned rabbit model to evaluate the effect of oat β-glucan on growth and intestinal development and to determine whether this effect was related to gut microbiota and related metabolites using integrated omics analyses, including 16S rDNA sequencing analysis and metabolomics analysis. Our study will provide a better understanding of the role of oat β-glucan in intestinal function, meanwhile, it is no longer limited to local gut microbiota metabolism, but expanded to host metabolism. The study also helps to clarify the effect of oat β-glucan on the growth of weaned rabbits, promote the application of oat β-glucan as a non-antimicrobial alternative feed additive in rabbit or other young animals rearing.

## 2 Materials and methods

### 2.1 Animals, management, and experiment design

**FIGURE 1:**
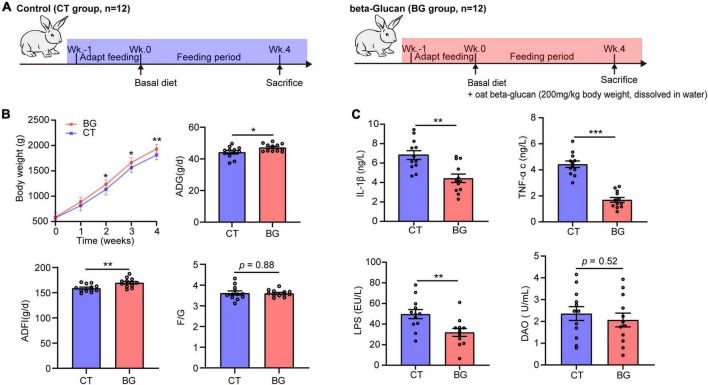
**(A)** Experimental setup for control (CT) and oat β-glucan (BG) groups. **(B)** Oat β-glucan improved growth performance. ADG, average daily gain; ADFI, average daily food intake; F/G, feed-to-gain ratio. Body weight (BW) data are presented as means ± SD. **(C)** Oat β-glucan decreases serum TNF-α, IL-1β, LPS, and DAO contents. TNF-α, tumor necrosis factor-α; IL-1β, interleukin-1β; LPS, lipopolysaccharide; DAO, diamine oxidase. *p < 0.05, **p < 0.01, and ***p < 0.001.

This study was conducted in strict accordance with the guidelines for the Care and Use of Laboratory Animals of China and all procedures were approved by the Animal Care and Use Committee of Sichuan Agricultural University (No. DYY-2018203039). Twenty-four 3 week old weaned female New Zealand white rabbits were obtained from Chengdu Dossy Experimental Animals Co., Ltd., (Chengdu, China) and individually housed in cages at a controlled temperature (22 ± 2°C), with a 12 h light/dark period in the Laboratory Animal Center of Sichuan Agriculture University (Yaan, China), rabbits have free access to water and standard diet. During 1 week of acclimatization, the rabbits were fed a basal diet. The rabbits were randomly divided into two groups after acclimation. Rabbits in the control group (*n* = 12, CT group) and oat β-glucan group (*n* = 12, BG group) were fed a normal basal diet, and rabbits in the BG group were also administered oat β-glucan (200 mg/kg BW, dissolved in 20 ml drinking water), according to the dosage of Zhang et al. (2012). Rabbits would be supplemented with fresh water after drinking up the water dissolved with oat β-glucan every day. The feeding period lasted for 4 weeks (Figure 1A). Oat β-glucan (88.69% purity on an air-dry basis; batch number SF-OGBO-210616) was purchased from Baixing Biological Technology Co., Ltd., Shandong, China.

At the end of the experiment at week four, fasting blood samples (6 h fasted) were collected from the rabbit hearts, followed by centrifuging at 1,500 × g for 10 min at 4°C, and the serum samples were isolated and frozen at −80°C for further analysis. Rabbits were anesthetized with an intravenous injection of 10% urethane (3 ml/kg BW) and euthanized by cervical dislocation. Colonic contents from the gut of each rabbit was collected into germ-free frozen tubes and stored at −80°C for intestinal microbiota analysis and metabolomics analysis. Six rabbits were randomly selected from each group, and distal colon tissues (approximately 3 cm) were collected, immediately flushed with cold saline, and fixed in 4% paraformaldehyde solution for histological analysis.

### 2.2 Food intake and growth

Body weight (BW) and food consumption were recorded weekly throughout the experiment, and the average daily gain (ADG), average daily food intake (ADFI), and the feed to gain ratio (F/G) were calculated for each week and the whole feeding period.

### 2.3 Measurement of serum biochemical indices

The concentrations of tumor necrosis factor-α (TNF-α), interleukin-1β (IL-1β), and LPS in the serum of each rabbit were measured using commercially available test kits from Nanjing Jiangcheng Bioengineering Institute, Nanjing, China (#H052-1-1, #H002-1-1, and #H255-1-1, respectively). Serum diamine oxidase (DAO) was measured using a commercial kit from Beijing Solarbio Science and Technology Co., Ltd., Beijing, China (#BC1285).

### 2.4 Histological analysis

After fixation in 4% paraformaldehyde solution for 48 h, the collected colon tissues were embedded in paraffin, sectioned at 5 μm using a rotary microtome (Leica RM2235, Germany), and stained with hematoxylin and eosin and periodic acid–Schiff. All the sections were visualized using a microimaging system (Leica DM2000, Germany). Villous height, crypt depth, muscular layer width, and mucin area proportion were measured using Image-Pro Plus version 6.0 (Image-Pro Plus software, Media Cybernetics, USA), and the villous height to crypt depth ratio was calculated. Ten microscopic fields were randomly selected from each section of the testes.

### 2.5 Intestinal microbiota analysis

The colonic content microbial genome was obtained using a Magnetic Soil and Stool DNA Kit (Tiangen Biotech (Beijing) Co., Ltd., Beijing, China), and the hypervariable V3--V4 region of the bacterial 16S rRNA gene was sequenced using primers (338F:5′-ACTCCTACGGGAGGCAGCA-3′; 806R:5′-GGACTACHVGGGTWTCTAAT-3′) on the Illumina Novaseq 6000 system at Shanghai Biotree Biotech Co., Ltd., Shanghai, China. The 16S rDNA gene sequencing data were merged using FLASH software (version 1.2.7) and filtered using Trimmomatic software (version 0.33), after which Cutadapt software (version 1.9.1) was used to identify and remove the primer pairs to acquire clean reads. UCHIME software (version 4.2) was used to identify and remove chimeric sequences to obtain effective reads. Operational taxonomic units (OTUs) were clustered with an identity threshold of 97% using the Usearch software (version 10.0). A representative sequence for each OTU was selected, and a classify-sklearn naive Bayes taxonomy classifier was employed to annotate the taxonomic information for each representative sequence against the SILVA database (Release 132).^1^ Rarefaction curve analysis was performed using Mothur software.^2^ QIIME 2 was^3^ used to evaluate the alpha diversity index (Chao1 and Shannon) and analyze beta diversity (non-metric multidimensional scaling, NMDS) using unweighted UniFrac methods. Bioinformatics analysis was performed using the online platforms Wekemo Bioincloud^4^ and Omicstudio.^5^

### 2.6 Untargeted metabolomics analysis

Liquid chromatography-mass spectrometry based metabolomics was used to obtained serum and colon metabolic profiles. The extraction and measurement procedures were described in detail in the Supplementary material. The metabolites of the serum samples were identified according to Luo Z. Z. et al. (2019). Metabolites of colonic content samples were identified based on MS/MS spectra and matched against an in-house MS2 database (BiotreeDB, version 2.1). Principal component analysis (PCA) was conducted to evaluate the differences between samples within and between groups. Variable importance in the projection (VIP) values in the orthogonal partial least squares discrimination analysis (OPLS-DA) model and *p* values from the Student’s *t*-test were implemented to screen the discriminatory components. Metabolites with VIP > 1 and *p* < 0.05 were set as significantly differential metabolites, while those with VIP > 1, and 0.05 ≤ *p* < 0.1 were considered as metabolites with different trends. The differential metabolites were visualized, and the Kyoto Encyclopedia of Genes and Genomes (KEGG) pathway analysis was performed using MetaboAnalyst 5.0.^6^

### 2.7 Intestinal *s*hort chain fatty acids analysis

Six colonic content samples were randomly selected from each group, and concentrations of SCFAs in them were determined using a gas chromatography mass spectrometer (Agilent 7890A/5975C, USA) at Shanghai Applied Protein Technology Co., Ltd., Shanghai, China. Briefly, a total of 30 mg colonic content was thawed, suspended in 900 μL of 0.5% phosphoric acid, and vortexed for 2 min. Each sample was centrifuged at 14,000 × g for 10 min and the phases were separated. The supernatant (800 μL) was then transferred into a 2 ml centrifuge tube and mixed with 800 μL of ethyl acetate to extract SCFAs. After centrifugation at 14,000 × g for 10 min, 600 μL of the upper layer was taken, 25 μL of 4-methylvaleric acid (500 μM) was added as an internal standard, and 1 μL of the mixture was injected into a sample injection bottle for further analysis.

All samples were analyzed with a 10:1 split ratio and separated using a gas chromatograph (Agilent DB-WAX, USA; capillary column: 30 m × 0.25 mm ID × 0.25 μm) at an oven temperature of 90°C, increased to 120°C at a rate of 10°C/min, then increased to 180°C at 5°C/min, finally at a rate of 25°C/min to 250°C, and held for 2 min; the flow rate of hydrogen was 1 ml/min. SCFA concentration was determined using the external standard method with corresponding standards and expressed in mg/g of colonic content. The total SCFA concentration was calculated as the sum of acetic, propionic, butyric, valeric, isovaleric, and isobutyric acids.

### 2.8 Statistical analysis

Differences between groups were evaluated by two-tailed unpaired Student’s *t*-tests using the SPSS software (version 17.0, Chicago, USA). Two-way ANOVA analysis and graph drawing were conducted using GraphPad Prism software (version 8.0.2, La Jolla, CA, USA) unless otherwise specified. The significance threshold was set at *p* < 0.05; trends were declared at 0.05 ≤ *p* < 0.10. Correlation analyses among the screened differential metabolites, SCFAs, biochemical indices, and the relative intestinal microbiota abundance were performed by Spearman’s correlation analysis using the R software. Data are presented as mean ± standard error unless otherwise indicated.

## 3 Results

### 3.1 Oat β-glucan improved growth performance in weaned rabbits

The BW, ADG ADFI, and F/G are shown in Figure 1B. Rabbits supplemented with oat β-glucan showed an increase in BW, which was significantly increased after feeding for 2 weeks (*p* < 0.05) and 3 weeks (*p* < 0.05), and significantly increased at 4 weeks (*p* < 0.01). Additionally, oat β-glucan accelerated ADG and ADFI (*p* < 0.05), but did not affect F/G (*p* > 0.05). In detail, rabbits in the BG group had a higher ADG (*p* < 0.05) after feeding for 1 week and had much more AFDI (*p* < 0.05) at 3 weeks. Oat β-glucan also significantly reduced the F/G ratio (*p* < 0.05) after feeding for 1 week. Moreover, feeding time significantly affected growth performance traits (except for F/G) (*p* < 0.05), whereas the interaction effect between β-glucan and feeding time was not significant on BW, ADG, ADFI, and F/G (*p* > 0.05, Supplementary Table 1).

### 3.2 Oat β-glucan altered serum biochemical indices and intestinal microstructure

Compared to the control group, oat β-glucan markedly reduced serum TNF-α, IL-1β, and LPS concentrations (*p* < 0.001), while the serum DAO concentration did not differ between treatments (*p* = 0.52), but showed a slight decrease in the BG group (Figure 1C). Oat β-glucan considerable increased the intestinal muscular layer width, villus height, villous height, crypt depth ratio, and mucin area proportion, and decreased crypt depth (*p* > 0.05, Supplementary Table 2 and Supplementary Figure 1).

### 3.3 Oat β-glucan modulated intestinal microbiota

**FIGURE 2:**
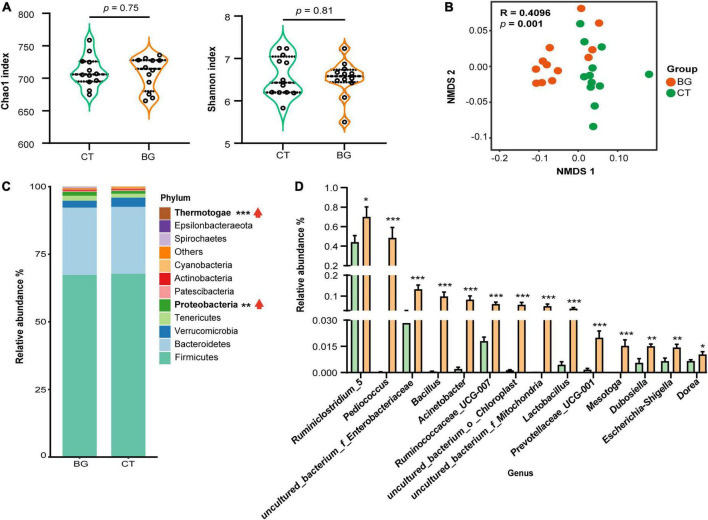
Oat β-glucan altered gut microbiota abundance of weaned rabbits. **(A)** Alpha-diversity of bacterial communities in colon. **(B)** Non-metric multidimensional scaling (NMDS) analysis plots of bacteria communities in oat β-glucan (BG) and control (CT) groups. **(C)** The abundance of gut microbiota at the phylum level. **(D)** Differential gut bacteria at the genus level. *p < 0.05, **p < 0.01, and ***p < 0.001. The upward red arrow indicates the relative abundance of this phylum was increased after feeding oat β-glucan.

After removing the chimeric sequences, 1,877,535 effective reads were generated from 24 colonic content samples, with an average of 78,230 effective reads per sample. Venn plot analysis showed that there were 810 OTUs shared between the two groups, while only six and five OTUs were uniquely present in the BG and CT groups, respectively (Supplementary Figure 2A). The rarefaction curve reached a plateau at a sequencing depth of 20,000, indicating that the sequencing depth was sufficient to represent most bacterial OTUs (Supplementary Figure 2B). The Chao1 and Shannon index values, used to evaluate community richness and diversity in each microbiome sample, were not significantly different between the two groups (*p* > 0.05, Figure 2A), whereas the NMDS by unweighted UniFrac distance analysis demonstrated that the BG group was distant from the CT groups (Figure 2B), indicating that oat β-glucan did not significantly change the bacterial diversity but altered the overall microbiota structure.

We further analyzed the composition and structure of gut microbial populations at the phylum and genus levels. According to the phylum assignment results, Firmicutes, Bacteroidetes, Verrucomicrobia, Tenericutes, and Proteobacteria were the most dominant bacteria, accounting for over 95% of the taxonomic groups identified. Compared to the CT group, the relative Proteobacteria and Thermotogae abundances were significantly increased (Figure 2C, *p* < 0.01). Inter-group comparisons of taxonomic profiles at the genus level showed that the BG group exhibited a higher relative abundance of *Ruminiclostridium_5*, *Pediococcus*, *uncultured_bacterium_f_Enterobacteriaceae*, *Bacillus*, *Acinetobacter*, *Ruminococcaceae_UCG-007*, *uncultured_bacterium_o_Chloroplast*, *uncultured_bacterium _f_Mitochondria*, *Lactobacillus*, *Prevotellaceae_UCG-001*, *Mesotoga*, *Dubosiella*, *Escherichia-Shigella*, and *Dorea* as compared to the CT group (Figure 2D, *p* < 0.05).

### 3.4 Oat β-glucan changed intestinal metabolism

To further explore the effects of oat β-glucan supplementation on intestinal microbiota metabolism, we performed an untargeted metabolome assay. The PCA score plot showed that the BG and CT groups were clearly clustered into two separate groups in both the positive and negative ion modes (Supplementary Figures 3A, B), indicating that oat β-glucan might modulate gut microbiota metabolism. The OPLS-DA score plot exhibited a similar trend of sample distribution, with the permutation tests for the model of each group showing a Q^2^ intercept of <0.05, indicating that there was no overfitting (Supplementary Figures 3C–F). The results indicated that the established OPLS-DA model was suitable. Then, the VIP value obtained from the OPLS-DA mode and the *p*-value from Student’s *t*-test were used to screen potential differential components. In total, 264 metabolites that differed between the BG and CT groups were screened (VIP > 1, *p* < 0.1). We then determined the origin of these differential metabolites using MetOrigin^7^, and found 17 bacterial metabolites, 15 bacteria–host metabolites, 2 host-specific metabolite, and 230 others (27 drug-related metabolites, 153 food-related metabolites, 1 environment metabolite, and 49 unknown metabolites), as shown in Supplementary Figure 3G.

**FIGURE 3:**
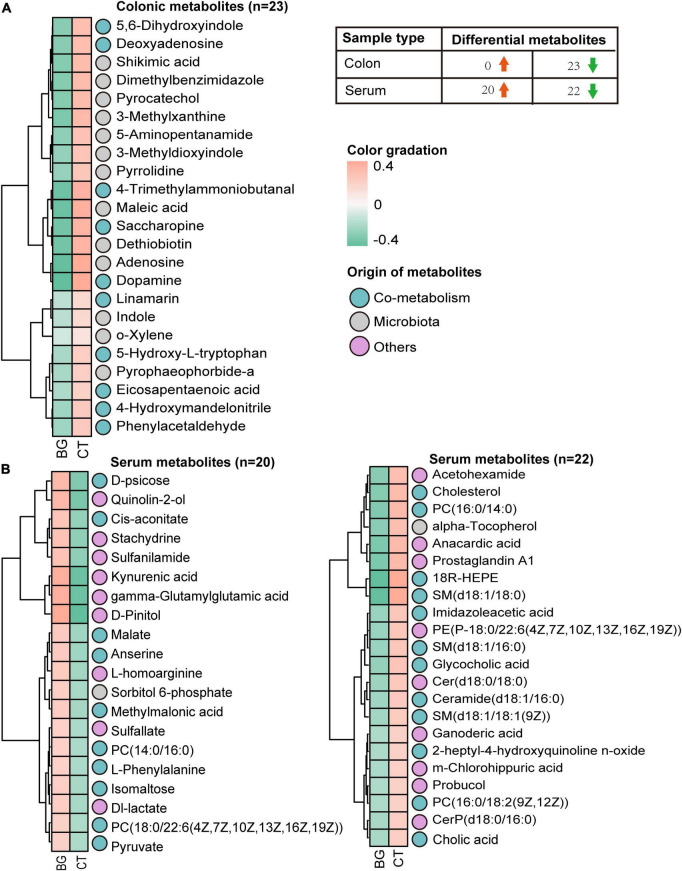
The heatmap. **(A)** Heatmap visualization of the colonic metabolites that differed between the oat β-glucan (BG) and control (CT) groups. **(B)** Heatmap visualization of the serum metabolites that differed between the oat BG and CT groups.

Next, we focused on the differential metabolites from the host, microbiota, and shared by both and mapped them in the KEGG database^8^ for KEGG second-grade pathway analysis. Finally, 23 metabolites were identified (Supplementary Table 3), including lysine derivatives (saccharopine, 5-aminopentanamide, and 4-trimethylammoniobutanal), tyrosine derivatives (dopamine, 5, 6-dihydroxyindole, and maleic acid), tryptophan derivatives (5-hydroxy-L-tryptophan, indole, and 3-methyldioxyindole), phenylalanine derivatives (phenylacetaldehyde), and purine metabolites (deoxyadenosine and adenosine). The levels of all these 23 metabolites, which were mainly involved in lysine degradation, purine metabolism, and aromatic amino acid (AAA) metabolism according to KEGG metabolic enrichment pathway analysis (Supplementary Figure 3H), were found to be decreased in the BG group (Figure 3). Taken together, these data suggest that supplementation with oat β-glucan can alter intestinal metabolic profiles in weaned rabbits.

### 3.5 Oat β-glucan changed serum metabolism

Microbial metabolites can be absorbed across the host gut, are measurable in the host circulation, and serve as signaling molecules and substrates for metabolic reactions that affect host physiology (Krautkramer et al., 2021). Therefore, we analyzed the serum metabolites of rabbits to assess whether local intestinal metabolic alterations would influence the serum metabolism profiles. The OPLS-DA mode exhibited a significant separation of clusters between the BG and CT groups, and the permutation tests for the model of each group showed a Q2 intercept of <0.05 (Supplementary Figures 4A–D), suggesting that the OPLS-DA model is suitable for screening differential metabolites. Compared with the serum metabolomics of the controls, we identified 42 differential metabolites (VIP > 1, *p* < 0.1), of which 26 were considered to be significantly expressed (VIP > 1, *p* < 0.05). Among them, two were bacterial metabolites, 19 were bacteria–host metabolites, and 21 belonged to others (Supplementary Figure 4E). KEGG metabolic enrichment pathway analysis found these metabolites mainly involved in carbohydrate, lipid, and amino acid metabolism (Supplementary Figure 4F). Furthermore, according to the Human Metabolome Database (HMDB),^9^ classification, seven belonged to carboxylic acids and derivatives, six metabolites belonged to sphingolipids, five belonged to organooxygen compounds, five belonged to glycerophospholipids, three belonged to quinolines and derivatives, three belonged to steroids and steroid derivatives, three belonged to benzene and substituted derivatives, two belonged to hydroxy acids and derivatives, two belonged to fatty acids, two belonged to prenol lipids, and the rest belonged to others. Heatmap analysis revealed 22 differentially expressed metabolites that were upregulated and 20 that were downregulated (Figure 3 and Supplementary Table 4).

### 3.6 Oat β-glucan promoted intestinal SCFA fermentation

**FIGURE 4:**
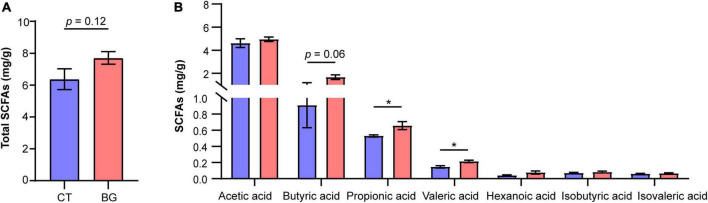
**(A)** Total concentration of short-chain fatty acids (SCFAs). **(B)** The concentration of acetic, propionic, butyric, valeric, isovaleric, and isobutyric acids. *p < 0.05.

Previous studies found that β-glucan was utilized by gut microbiota to produce SCFAs; therefore, we also measured concentrations of SCFAs in the colonic content. The results showed that after supplementation with oat β-glucan for 4 weeks, the total concentration of SCFAs increased compared to that in the control group, but the difference was not significant (*p* = 0.12, Figure 4A). Particularly, propionic and valeric acid concentrations increased significantly (*p* < 0.05) in the BG group, and butyric acid also demonstrated an increasing tendency (*p* = 0.06, Figure 4B).

### 3.7 Correlation analyses among phenotype, intestinal microbiota, and related metabolites

**FIGURE 5:**
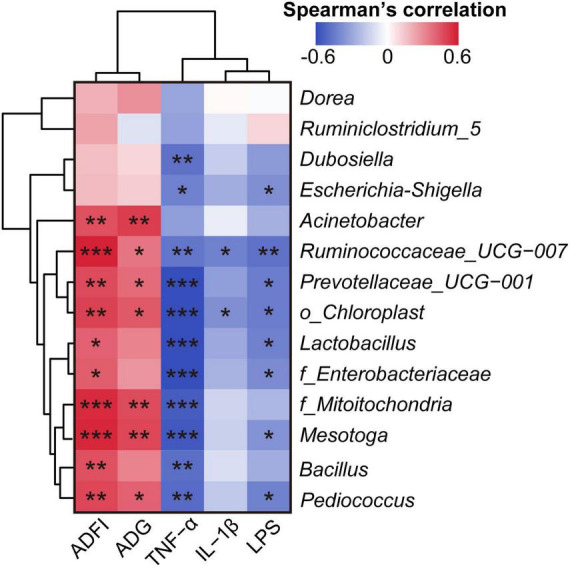
Spearman’s correlation analysis of clinical phenotype indices and the relative abundance of differential bacteria at the genus level. *p < 0.05, **p < 0.01, and ***p < 0.001. The Spearman’s correlation coefficient of significant correlations >0.4 (or <–0.4).

Spearman’s correlation analysis between the microbiome and clinical phenotype indices (LPS, IL-1β, TNF-α, ADG, and ADFI) revealed that most of these differential gut bacteria at the genus level negatively correlated with LPS, IL-1β, and TNF-α, and positively correlated with ADG and ADFI (Figure 5).

Spearman’s correlation analysis between the microbiome and related differentially expressed gut metabolites revealed that most of these metabolites negatively correlated with the gut bacteria. Particularly, dopamine and saccharopine showed strong correlations with most of the microbial taxa (*p* < 0.05, Supplementary Figure 5). Correlation analysis of the gut bacteria and serum metabolites showed that gut bacteria negatively associated with metabolites that involved in lipid metabolism, and positively correlated with metabolites which participated in carbohydrate and amino acid metabolism (Supplementary Figure 6). Then between layers correlation of gut metabolites and serum metabolites calculated, the results showed that the correlation between the two layers was not strong (Supplementary Figure 7).

**FIGURE 6:**
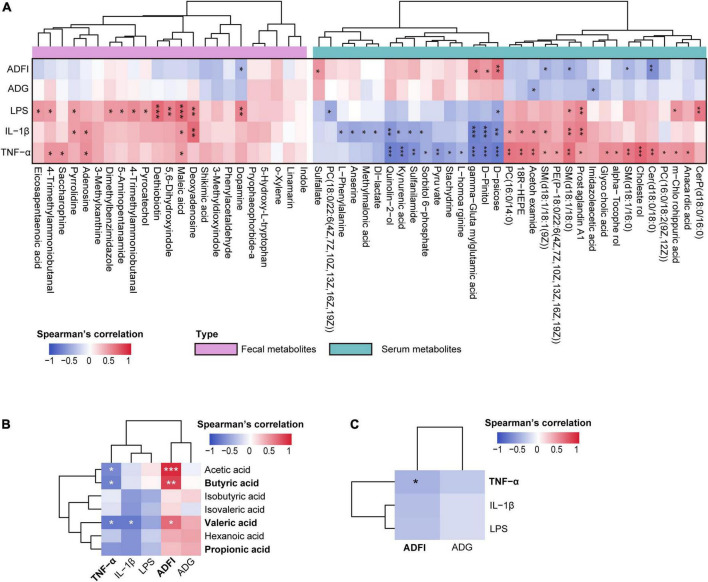
**(A)** Spearman’s correlation analysis between differential metabolites (colonic contents and serum) and clinical phenotype indices. **(B)** Spearman’s correlation analysis between the colonic short-chain fatty acids (SCFAs) and clinical phenotype. **(C)** Spearman’s correlation analysis between the inflammatory-related indices (tumor necrosis factor-α [TNF-α], interleukin-1β [IL-1β], and lipopolysaccharide [LPS]) and growth performance-related indices (average daily gain [ADG], average daily food intake [ADFI]). *p < 0.05, **p < 0.01, and ***p < 0.001. The spearman’s correlation coefficient of significant correlations > 0.4 (or <–0.4).

Further correlation analysis of gut metabolites and clinical phenotype indices suggested that most of these metabolites significantly positively correlated with LPS, IL-1β, and TNF-α levels (*p* < 0.05, Figure 6A).

Spearman’s correlation analysis between serum metabolites and clinical phenotype indices was performed, and significant correlations were present in a heatmap (Figure 6A). The results showed that LPS, IL-1β, and TNF-α significantly correlated with most serum metabolites (*p* < 0.05); however, ADG and ADFI showed no significant correlations with most serum metabolites (*p* > 0.05). Interestingly, inflammation-related indices (LPS, IL-1β, and TNF-α) and growth-related indices (ADG and ADFI) showed opposite relationships with serum metabolites.

Spearman’s correlation analysis between SCFAs and the microbiome revealed that colonic propionic acid and valeric acid concentrations exhibited a strong positive correlation with most of these bacteria (*p* < 0.05). Additionally, acetic and butyric acids positively correlated with *Ruminiclostridium_5* and *uncultured_bacterium_o_Chloroplast* (*p* < 0.05), whereas isobutyric acid negatively correlated with *Dubosiella* (*p* < 0.05, Supplementary Figure 8). These results suggest that changes in microbiome may promote the production of SCFAs in weaned rabbit guts.

Short-chain fatty acids (SCFAs) not only play a role in maintaining intestinal homeostasis but also circulate in blood and directly affect metabolism or function of peripheral tissues (van der Hee and Wells, 2021); thus, we performed Spearman’s correlation analysis to identify the associations between SCFAs and clinical phenotype indices. Consequently, a negative correlation between inflammation-related indices and SCFAs was observed. Particularly, serum TNF-α significantly negatively correlated with acetic, butyric, and valeric acids, and IL-1β significantly negatively correlated with valeric acid (*p* < 0.05). Conversely, ADG and ADFI were positively correlated with SCFAs, of which ADFI significantly positively associated with acetic, butyric, and valeric acids (*p* < 0.05, Figure 6B). These results prompted us to identify the links between these inflammatory and growth-related indices and found that they negatively correlated; indeed, TNF-α significantly negatively correlated with ADFI (*p* < 0.05, Figure 6C). Taken together, these findings indicate that an altered gut microbiome changes gut metabolism and promotes the production of SCFAs, and further influences serum metabolic profiles, thus regulating the production of inflammatory cytokines and improving growth performance.

## 4 Discussion

In this study, we investigated the effects of oat β-glucan supplementation on weaned rabbits. After 4 weeks of oat β-glucan treatment, a significant increase in BW, daily gain, and food intake were observed. The effects of different β-glucan sources on growth performance have been previously reported. Luo J. et al. (2019) found that a basal diet supplemented with 100 mg/kg bacterial β-glucan improved ADG in weaned piglets. Oral administrated with 75 mg/kg oat β-glucan also increased BW in pre-weaning dairy calves (Luo et al., 2022). Additionally, oral administration of pharmaceutical grade 1, 3 β-glucan at doses of 0.25 and 0.5 ml/L of drinking water significantly accelerated BW gain and reduced the feed conversion ratio in 6-week-old rabbits (Abo Ghanima et al., 2020). Claves supplemented with 1 g/day seaweed β-glucan had lower food intake and ADG, which was probably due to excessive β-glucan addition (McDonnell et al., 2019). However, to the best of our knowledge, limited research has focused on its role in promoting growth, particularly in young animals. Our results suggest that feeding oat β-glucan can improve growth performance; this might not only reflect the feeding amount of oat β-glucan in this work is suitable, but also provide a basis for its application in young animal rearing in the future.

We also observed a decrease in serum LPS levels. LPS is a component of the gram-negative bacterial cell wall and is generally considered as a trigger inducing localized or systemic inflammation by increasing the production of proinflammatory cytokines, such as IL-1β, interleukin-6 (IL-6), and TNF-α, and excess LPS leaked from the gut to the bloodstream usually reflects gut barrier integrity damage (Cox and Blaser, 2013; You et al., 2013; Guo et al., 2015). Therefore, the decrease in serum LPS has a causative role in the decrease of serum IL-1β and TNF-α levels in this study.

Numerous studies have shown a strong correlation between intestinal function and growth performance in animals (Zou et al., 2022). Therefore, we measured the colon microstructure and found that oat β-glucan could promote intestinal development to some extent. Like LPS, DAO is usually located at the upper villi of the small intestinal mucosa and enters the circulation when epithelial cells are damaged (Zhang et al., 2020). Thus, both serum DAO and LPS levels can reflect gut epithelial permeability and integrity. In this study, a decrease in serum DAO and LPS concentrations were found in the BG group, which might indicate that oat β-glucan also improved the intestinal epithelial barrier function. TNF-α is mainly produced by inflammatory cells such as macrophages and activated T-cells, and initiates inflammatory responses *via* the nuclear factor-κB (NF-κB) and mitogen-activated protein kinase signaling pathways (Oceandy et al., 2019). It has also been shown to induce apoptosis and inflammatory responses in intestinal epithelial cells and impair the intestinal barrier through cytoskeletal rearrangement and tight junction protein expression regulation. Previous studies found that IL-1β increases in the intestinal mucosa under inflammatory conditions, and like TNF-α, it impairs the intestinal tight junction barrier through decreases in occludin and cytoskeletal rearrangement (Suzuki, 2013). In this regard, we assumed that oat β-glucan might improve intestinal barrier function by regulating TNF-α and IL-1β expressions. In brief, oat β-glucan may improve the growth performance of weaned rabbits by enhancing gut function, not only promoting intestinal development but also protecting barrier integrity.

Accumulating evidence demonstrates that gut microbiota and its catabolites are closely associated with gut function (Valdes et al., 2018). A previous study found that β-glucan ingestion suppressed inflammation and improved colonic mucosal barrier function in ulcerative colitic mice, which was mediated by gut-derived SCFAs and intestinal microbial metabolic biomarkers (Bai et al., 2021b). In this study, oat β-glucan supplementation significantly altered the gut microbiota structure and composition in weaned rabbits. Particularly, at the genus level, the relative abundance of some beneficial bacteria, such as *Lactobacillus*, *Prevotellaceae_UCG-001*, *Pediococcus*, and *Bacillus*, significantly increased in the BG group. Among these, *Lactobacillus*, *Pediococcus*, and *Bacillus* are the three most common probiotics. *Lactobacillus* is implicated with beneficial effects in preventing and reducing inflammation-related diseases, such as inflammatory bowel disease (IBD) and non-alcoholic fatty acid liver diseases, accompanied by gastrointestinal barrier function enhancement and a decrease in pro-inflammatory cytokine levels (such as IL-6 and IL-1β) (Azad et al., 2018; Aghamohammad et al., 2022). A previous study demonstrated that oat β-glucan increased the population of *Lactobacillus* during *in vitro* fermentation when inoculated with mouse fecal microbiota (Jayachandran et al., 2018). Additionally, dietary supplementation with β-glucan from *Candida glabrata* also increased the relative abundance of *Lactobacillus* in colitic mice (Charlet et al., 2018). Similar to *Lactobacillus*, *Pediococcus* also exhibits beneficial effects in non-alcoholic fatty acid liver diseases by modulating the gut microbiome and inflammatory pathways (Lee et al., 2020). A recent study demonstrated that the increase in *Pediococcus* caused by feeding selenium-enriched *Pediococcus acidilactici* MRS-7 contributed to intestinal barrier function restoration and alleviation of the inflammatory response and oxidative stress in jejunum-injured mice (Chen et al., 2022). *Bacillus* strains have been widely used as potential probiotics to combat pathogen invasion and improve growth performance (Wang et al., 2020; Sam-On et al., 2022). Zou et al. (2022) found that dietary supplementation with *Bacillus subtilis* decreased proinflammatory cytokine expression and improved antioxidative status and intestinal barrier integrity by regulating the intestinal microenvironment and related microbiota composition in laying hens. As one of the most common members of intestinal bacteria, accumulating studies have presented a lower relative abundance of *Prevotellaceae_UCG-001* in the gut of animals with colitis and have rebalanced it after colitis alleviation. A previous report also showed that metabolites of *Prevotellaceae UCG-001* can activate the adenosine monophosphate (AMP)-activated protein kinase signaling pathway to improve gut health (Song et al., 2019). In the BG group, we also observed an increased abundance of *Ruminococcaceae_UCG-007* and *Ruminiclostridium_5*, which have rarely been reported previously. *Ruminococcaceae* is generally considered a beneficial bacterium colonized in the cecum and colon and can degrade various polysaccharides and fibers to produce SCFAs (Huang et al., 2021), it also presents an effect to reduce intestinal inflammation (Hu et al., 2021). Moreover, findings from prior research showed that the increase of *Ruminiclostridium_5* was associated with positive health states, whereas reduced levels are associated with negative health states. For instance, a prebiotic diet produced a rapid and stable increase in the relative abundance of *Ruminiclostridium_5* in mice with the chronic disruption of rhythm (Thompson et al., 2021). *Ruminiclostridium_5* was also abundant owing to the alleviation of DSS-induced colitis (Shao et al., 2020). These results may explain the higher SCFA content in the BG group. These genera are SCFA-producing bacteria, as previously reported. SCFAs can modulate the expression of tight junction proteins to maintain gut epithelial barrier integrity and protect gut epithelium by enhancing mucin 2 expression, modulating oxidative stress, and immune response (Feng et al., 2018). Therefore, SCFAs play a crucial role in the maintenance of intestinal homeostasis. Moreover, *Lactobacillus* and *Ruminiclostridium_5* were reported to produce butyric acid, which can nourish and protect the intestinal epithelium (Fu et al., 2019; Shao et al., 2020).

It is also worth noting that oat β-glucan increased the abundances of phyla Proteobacteria and genus *uncultured_bacterium_f_Enterobacteriaceae*, *uncultured_bacterium_f_Mitochondria*, *Acinetobacter*, and *Escherichia-Shigella* (phylum Proteobacteria), which are often considered as pathogens. This is consistent with a previous report that Proteobacteria were adapted for food rich in non-starch polysaccharides such as xylan and cellulose. They speculated that this might be due to increased fiber intake that decreases fat and protein digestibility, which ultimately increases the amount of fermentation substrates available for various gut microorganisms, such as Proteobacteria (Tian et al., 2017). A previous study also found that dietary supplementation with oat-derived β-glucan increased the relative abundance of Betaproteobacteria, Gammaproteobacteria (including *Enterobacteriaceae*), and Deltaproteobacteria in BALB/c mice (Luo et al., 2017). Taken together, these data indicate that oat β-glucan might enhance intestinal function by increasing the abundance of beneficial gut microbiota communities and their metabolites, SCFAs.

Interestingly, except for SCFAs, correlation analysis also indicated that these beneficial bacteria have profound relationships with other intestinal metabolites. In this study, the levels of saccharopine and the two other metabolites, 5-aminopentanamide and 4-trimethylammoniobutanal were lower in the BG group, and KEGG pathway analysis revealed that they participated in the lysine degradation pathway. Lysine is an essential amino acid, and its degradation has two distinct pathways. In the saccharopine pathway, lysine is converted to saccharopine *via* α-aminoadipic semialdehyde synthase, whereas in the pipecolate pathway, lysine is first converted to 5-aminopentanamide (Crowther et al., 2019). Saccharopine accumulation induces mitochondrial dysfunction in mammals (Leandro and Houten, 2019), and has also been shown to participate in gluconeogenesis through its role in the propionyl-CoA synthesis pathway and tricarboxylic acid cycle *via* the acetyl-CoA production pathway (Safi-Stibler et al., 2020). In this regard, the decrease in saccharopine might partially reflect an alteration in the way gut microbiota acquires energy. Additionally, higher 5-aminopentanamide content in the gut has been strongly associated with IBDs, such as ulcerative colitis and Crohn’s disease (Lin et al., 2010; Feng et al., 2020). Besides lysine degradation, we observed alterations in the AAA metabolism. Research has shown that increasing carbohydrate availability in the hindgut leads to microbial AAA metabolism inhibition and an increase in systemic AAA availability in piglets (Gao et al., 2019).

In line with this previous study, oat β-glucan also decreased the intestinal contents of AAA metabolites in weaned rabbits in this study, and the serum contents of l-phenylalanine and kynurenic acid (formed from tryptophan) were higher in the BG group. An explanation for this occurrence is that the fiber supplementation promotes intestinal microbiota to preferentially ferment easily fermented carbohydrates, but not proteins, thus decreasing the production of amino acid (AAA and lysine) metabolites (Gao et al., 2018). Meanwhile, there was no AAA elevation in the colon; therefore, we speculated that the upregulation of serum l-phenylalanine and kynurenic acid might reflect the enhancement of amino acid transport ability from the gut to the blood, as circulating AAA is mainly derived from gut absorption (Fernstrom, 2013). In contrast, the content of AAA biosynthesis precursor shikimic acid decreased in the BG group, which might suggest an increase in gut AAA biosynthesis, but this needs further investigation. Microbial AAA metabolites serve as signaling molecules that affect host physiology, such as by regulating intestinal epithelial cell homeostasis and immune cell response (Liu et al., 2020). In this study, we found that the microbial tryptophan metabolites indole and 3-methyldioxyindole were decreased in the BG group. 3-methyldioxyindole is an oxidation product of 3-methylindole (also called skatole) *in vivo* (Xu et al., 2020). Skatole is most probably a product in the colon and has been found to have a damaging effect on intestinal epithelial tissue activity and function after absorption but before detoxification (Yokoyama and Carlson, 1979). A previous study also suggested that indoles and skatoles exert an inhibitory effect on *CYP11A1* expression, which leads to a decrease in glucocorticoid production and further disturbs intestinal homeostasis (Mosa et al., 2016; Gao et al., 2018). Combined with the above results, we speculate that oat β-glucan supplementation enriched the available nutrient substrates in the intestine, which perhaps promotes the alteration of microbiota nutrition patterns and alters microbiota metabolism, further improving intestinal health by promoting the production of SCFAs and decreasing the levels of lysine and AAA-related metabolites.

Serum metabolic profile analysis revealed that oat β-glucan administration mainly altered host energy metabolism, including carbohydrate, lipid, and amino acid metabolisms. Correlation analysis suggested that altered serum metabolites were highly associated with inflammation-related indices LPS, IL-1β, and TNF-α. Most notably, sphingolipid metabolism changed significantly, which is consistent with a previous report that oat β-glucan treatment alters gut microbial sphingolipid metabolism in colitic mice (Bai et al., 2021b). Moreover, six sphingolipids, including SM (d18:1/16:0), ceramide (d18:1/16:0), SM [d18:1/18:1 (9Z)], Cer (d18:0/18:0), SM (d18:1/18:0), and CerP (d18:0/16:0), which were identified as serum metabolic biomarkers in this study, were all decreased in the BG group. Studies have suggested that TNF-α can initiate an inflammatory response through the acid and neutral sphingomyelinase pathways, which leads to increased ceramide production and eventually activates the pro-inflammatory transcription factor NF-κB (Sedger and McDermott, 2014). Similar to TNF-α, IL-1β exerts an effect on the induction of the ceramide-signaling pathway (Schneider et al., 2005). Ceramide is the center of sphingolipid synthesis and degradation and serves as a precursor for the synthesis of complex sphingolipids (Nixon, 2009). Additionally, TNF-α also activates phospholipase A_2_ enzymes, resulting in the metabolism of glycerophospholipids to arachidonic acid, which then degrades to prostaglandins *via* the cyclooxygenase pathway (Oceandy et al., 2019; Yuan et al., 2020). Based on these findings, we assumed that there is a close relationship between serum IL-1β and TNF-α concentrations, and serum sphingolipid and glycerophospholipid content; that is, oat β-glucan might inhibit the systemic inflammatory response by regulating serum sphingolipid and glycerophospholipid metabolism.

It is also worth noting that serum metabolites just showed slight correlations with growth performance related indices. However, both ADG and ADFI were negatively associated with LPS, IL-1β, and TNF-α, indicating that oat β-glucan may promote growth by inhibiting systemic inflammation development. Furthermore, SCFAs have been reported to exhibit anti-inflammatory effects by directly activating G-protein-coupled receptors (GPCRSs) and inhibiting nuclear class I histone deacetylases, leading to decreased proinflammatory cytokines, such as IL-6, interleukin-8, and TNF-α (Feng et al., 2018). Besides maintaining intestinal hemostasis and anti-inflammatory effects, gut-derived SCFAs are also involved in appetite control through hormonal and central effects. For instance, SCFAs can activate ghrelin-related signaling and inhibit insulin secretion by activating GPCR 3, which also crosses the blood-brain barrier to directly affect appetite-related neurons in the brain (Han et al., 2021). In this study, acetic, butyric, and valeric acids positively associated with ADFI, indicating that these play a pivotal role in promoting food intake. Moreover, a study found that high carbohydrate availability in the hindgut also led to an increase in AAA-derived neurotransmitters (serotonin and dopamine) in the hypothalamus of piglets (Gao et al., 2019), both of which are critical mediators of appetite control (Meguid et al., 2000; Han et al., 2021). Therefore, we assumed that, in rabbits, oat β-glucan administration might also stimulate food intake by modulating central serotonin and dopamine signaling, but the mechanisms deserve further investigation.

## 5 Conclusion

This study revealed that oat β-glucan can regulate gut microbiota structure and increase the abundance of potentially beneficial bacteria. Simultaneously, it can increase the concentration of SCFAs in the colon and decrease the concentrations of AAA and lysine-related metabolites. Oat β-glucan can also alter serum metabolic profiles and change the serum contents of 42 metabolites, including sphingolipids and glycerophospholipids. These results demonstrate the positive effect of oat β-glucan on lowering systemic inflammatory levels and promoting intestinal health, which further helps to improve growth performance. This study provides new insights into the prebiotic effects of oat β-glucan in a weaned rabbit model and promotes the application of oat β-glucan in young animals rearing. However, we have not explored the precise mechanism behind these effects. Further research is needed to clarify the interactions between SCFAs, AAA derivatives and intestinal epithelial tissue, and explore their role in the gut–brain axis.

## Data availability statement

The datasets presented in this study can be found in online repositories. The names of the repository/repositories and accession number(s) can be found below: NCBI-PRJNA901541.

## Ethics statement

This study was conducted in strict accordance with the guidelines for the Care and Use of Laboratory Animals of China and all procedures were approved by the Animal Care and Use Committee of Sichuan Agricultural University (No. DYY-2018203039).

## Author contributions

LM and ZL: conceptualization, methodology, investigation, writing – original draft, writing – reviewing and editing, and visualization. YXH: writing – original draft, writing – reviewing and editing, and visualization. YL and JG: methodology, investigation, writing – original draft, and writing – reviewing and editing. TZ, ZD, KY, XY, LS, and SY: investigation, writing – original draft, and writing – reviewing and editing. ZZ, YCH, and GP: writing – original draft and writing – reviewing and editing. XS: funding acquisition, project administration and supervision, writing – original draft, and writing – reviewing and editing. SC: conceptualization, writing – original draft, writing – reviewing and editing, funding acquisition, project administration, and supervision. All authors contributed to the article and approved the submitted version.

## Footnotes

1
http://www.arb-silva.de

2
http://www.mothur.org/

3
https://qiime2.org

4
https:/bioincloud.tech/

5
https://www.omicstudio.cn/tool

6
https://www.metaboanalyst.ca/

7
http://metorigin.met-bioinformatics.cn/

8
https://www.genome.jp/kegg/

9
https://hmdb.ca/
