# Supplementary material for: Modulating gut microbiota and metabolites with dietary fiber oat β-glucan interventions to improve growth performance and intestinal function in weaned rabbits
Source: Front Microbiol. 2022 Dec 15;13:1074036. doi: 10.3389/fmicb.2022.1074036 (PMC9798315; doi:10.3389/fmicb.2022.1074036)
Supplement: Supplementary file 1 [file Data_Sheet_1.PDF]

Supplementary Material

1 Supplementary Methods

Untargeted metabolomics analysis

Non-targeted metabolite analysis of the colonic contents was performed by Shanghai Biotree Biotech Co., Ltd., Shanghai, China. An ultra-high-performance liquid chromatography (UHPLC) system (Vanquish, Thermo Fisher Scientific) with a Waters ACQUITY UPLC BEH Amide column (2.1 mm × 100 mm, 1.7 μm) coupled to a Q Exactive HFX mass spectrometer (Orbitrap MS, Thermo) was used to analyze the colonic metabolic profiles. Colonic content sample pretreatment was prepared as follows: 25 mg of colonic content from each sample was weighed and 500 μL extract solution (methanol: acetonitrile: water = 2:2:1, v/v/v) with isotopically labeled internal standard mixture was added, then vortexed for 60 s, followed by homogenization at 35 Hz for 4 min and sonication for 5 min in an ice-water bath, which was repeated three times. After the samples were incubated for 1 h at -40 °C and centrifuged at 14,000 × g at 4 °C for 15 min. The resulting supernatant was transferred into a fresh glass vial for further analysis.

After sample pretreatment, the serum samples were separated using an ultra-high-performance liquid chromatography (UHPLC) system (1290 Infinity II, Agilent Technologies) incorporating a hydrophilic interaction chromatography column (2.1 × 100 mm, 1.7 µm; Waters). The mobile phase was 25 mmol/L of ammonium acetate and 25 mmol/L of ammonia hydroxide in water(A) and acetonitrile(B) in a gradient elution mode with a flow rate of 0.5 mL/min and an injection volume of 2 μL, the Gradient elution procedure was as follows: 0–0.5 min, 95% B; 0.5–7 min, 95% to 65% B; 7–8 min, 65% to 40% B; 8–9 min 40% B; 9–9.1 min 40% to 95% B; 9.1–12 min 95% B.; mass spectrometry analysis was performed using a triple time-of-flight (TOF) 6600+ system (AB SCIEX) equipped with an electrospray ionization source used in positive and negative ion modes. UHPLC-TOF-MS was performed by Shanghai Applied Protein Technology Co. Ltd. (Shanghai, China).

2 Supplementary Tables

Supplementary Table S1 Effect of oat β-glucan on growth performance of weaned rabbits

Item	Group	Time					*p*-value of two-way ANOVA		
		0W	1W	2W	3W	4W	Group	Time	Group×Time
BW/ kg	CT	0.57	0.81	1.13^a	1.56^a	1.81^A	0.00	0.00	0.35
	BG	0.58	0.88	1.23^b	1.66^b	1.93^B			
SEM		0.01	0.02	0.02	0.02	0.02			
ADG/g	CT	-	34.06^a	45.94	60.76	35.70	0.00	0.01	0.30
	BG	-	42.07^b	50.12	61.29	38.60			
SEM		-	1.64	1.17	1.04	1.51			
ADFI/g	CT	-	84.80	125.46	195.30^a	234.17	0.00	0.01	0.71
	BG	-	88.23	135.06	211.12^b	245.15			
SEM		-	2.39	4.31	3.71	4.67			
F/G	CT	-	2.60^a	2.76	3.24	6.73	0.00	0.38	0.34
	BG	-	2.12^b	2.71	3.47	6.55			
SEM		-	0.10	0.11	0.08	0.22			

[a,b]Means values with the column with different superscript letter was significant difference ($p < 0.05$), [A,B]Means values with the column with different superscript letter was highly significant difference ($p < 0.01$). SEM, standard error of mean.

Supplementary Table S2 Effect of oat β-glucan on the colon histomorphology of weaned rabbits

Item	CT	BG	SEM	*p*-value
Muscular layer width/ μm	249.53	262.34	15.10	0.36
Villus height/μm	257.65	274.65	8.62	0.17
Crypt depth/μm	145.03	139.31	9.75	0.39
Villous height / crypt depth	1.90	2.06	0.15	0.34
Proportion of mucin area/μm^2	0.25	0.31	0.021	0.15

SEM, standard error of mean.

Supplementary Table S3 Effect of oat β-glucan on the colon histomorphology of weaned rabbits

No.	Ions mode	Metabolite	Origin	VIP	Log2 (FC)	*p*-value	KEGG pathway
1	ESI+	Saccharopine	Co-Metabolism	1.07	-0.73	0.015	Lysine biosynthesis; Lysine degradation
2	ESI+	Dopamine	Co-Metabolism	1.25	-0.69	0.020	Tyrosine metabolism; Isoquinoline alkaloid biosynthesis; cAMP signaling pathway; Neuroactive ligand-receptor interaction; Gap junction; Synaptic vesicle cycle; Dopaminergic synapse; Prolactin signaling pathway; Bile secretion;
3	ESI+	4-Trimethylammoniobutanal	Co-Metabolism	1.32	-0.96	0.021	Lysine degradation
4	ESI+	5-Hydroxy-L-tryptophan	Co-Metabolism	1.03	-0.43	0.047	Tryptophan metabolism; Axon regeneration; Serotonergic synapse
5	ESI+	5,6-Dihydroxyindole	Co-Metabolism	1.21	-0.77	0.053	Tyrosine metabolism
6	ESI+	Deoxyadenosine	Co-Metabolism	1.10	-1.16	0.071	Purine metabolism; Nucleotide metabolism; ABC transporters
7	ESI+	4-Hydroxymandelonitrile	Co-Metabolism	1.01	-0.60	0.074	Cyanoamino acid metabolism
8	ESI+	Linamarin	Co-Metabolism	1.00	-0.48	0.075	Cyanoamino acid metabolism
9	ESI+	Phenylacetaldehyde	Co-Metabolism	1.03	-0.63	0.077	Phenylalanine metabolism; Styrene degradation
10	ESI+	Eicosapentaenoic acid	Co-Metabolism	1.03	-0.53	0.084	Biosynthesis of unsaturated fatty acids
11	ESI+	Dimethylbenzimidazole	Microbiota	1.56	-0.68	0.022	Riboflavin metabolism; Porphyrin metabolism; Biosynthesis of cofactors
12	ESI+	Dethiobiotin	Microbiota	1.06	-0.75	0.035	Biotin metabolism; Biosynthesis of cofactors
13	ESI+	5-Aminopentanamide	Microbiota	1.61	-0.85	0.036	Lysine degradation
14	ESI+	Pyrophaeophorbide a	Microbiota	1.41	-0.66	0.047	Porphyrin metabolism
15	ESI+	Pyrrolidine	Microbiota	1.22	-0.70	0.07	Nicotine degradation, pyrrolidine pathway, nicotine => succinate semialdehyde

Supplementary Table S3 Effect of oat β-glucan on the colon histomorphology of weaned rabbits

No.	Ions mode	Metabolite	Origin	VIP	Log2 (FC)	*p*-value	KEGG pathway
16	ESI+	Indole	Microbiota	1.41	-0.56	0.050	Tryptophan metabolism; Phenylalanine, tyrosine and tryptophan biosynthesis; Benzoxazinoid biosynthesis; Protein digestion and absorption
17	ESI+	3-Methyldioxyindole	Microbiota	1.13	-0.84	0.071	Tryptophan metabolism
18	ESI+	o-Xylene	Microbiota	1.24	-0.43	0.077	Xylene degradation; Degradation of aromatic compounds
19	ESI-	Adenosine	Co-Metabolism	1.07	-0.57	0.035	Purine metabolism; Nucleotide metabolism; ABC transporters; cGMP-PKG signaling pathway; cAMP signaling pathway; Sphingolipid signaling pathway; Neuroactive ligand-receptor interaction; Vascular smooth muscle contraction; Regulation of lipolysis in adipocytes; Renin secretion
20	ESI-	Maleic acid	Microbiota	1.42	-0.49	0.052	Tyrosine metabolism; Butanoate metabolism; Nicotinate and nicotinamide metabolism
21	ESI-	3-Methylxanthine	Microbiota	1.34	-0.46	0.068	Caffeine metabolism
22	ESI-	Shikimic acid	Microbiota	1.07	-0.42	0.089	Phenylalanine, tyrosine and tryptophan biosynthesis; Biosynthesis of amino acids; Biosynthesis of cofactors
23	ESI-	Pyrocatechol	Microbiota	1.07	-0.45	0.09	Chlorocyclohexane and chlorobenzene degradation; Benzoate degradation; Fluorobenzoate degradation; Dioxin degradation; Polycyclic aromatic hydrocarbon degradation; Naphthalene degradation; Aminobenzoate degradation; Degradation of aromatic compounds

KEGG, kyoto encyclopedia of genes and genomes; VIP, the variable importance in projection in orthogonal partial least-squares discrimination analysis; FC, fold change, Log2(FC) indicated the logarithmic function of fold change based on 2, Log2 (1) = 0.

Supplementary Table S4 Differential serum metabolites identified of oat β-glucan (BG) group vs. control (CT) group in the positive and negative mode.

No.	Ions mode	Adduct	Metabolite	HMDB Classification	VIP	Log2 (FC)	-Lg(*p*)	m/z	rt(s)
1	ESI+	[M+H]+	PC(18:0/22:6(4Z,7Z,10Z,13Z,16Z,19Z))	Glycerophospholipids	1.26	0.08	1.05	834.59	110.33
2	ESI-	[M+Cl]-	PC(16:0/18:2(9Z,12Z))	Glycerophospholipids	1.9	-0.05	1.02	792.53	154.45
3	ESI-	[M-H]-	18R-HEPE	Fatty Acyls	1.78	-0.21	4.14	780.53	51.26
4	ESI+	[M+H]+	PE(P-18:0/22:6(4Z,7Z,10Z,13Z,16Z,19Z))	Glycerophospholipids	4.56	-0.07	1.57	776.58	160.05
5	ESI-	[M+Cl]-	SM(d18:1/18:0)	Sphingolipids	1.7	-0.08	3.87	765.56	179.85
6	ESI-	[M+Cl]-	PC(14:0/16:0)	Glycerophospholipids	2.06	0.10	1.11	740.52	62.364
7	ESI+	[M+H]+	SM(d18:1/18:1(9Z))	Sphingolipids	4.98	-0.06	1.33	729.59	179.05
8	ESI+	[M+H]+	PC(16:0/14:0)	Glycerophospholipids	2.61	-0.10	2.39	706.53	152.13
9	ESI+	[M+H]+	SM(d18:1/16:0)	Sphingolipids	18.47	-0.06	1.60	703.57	181.44
10	ESI-	[M-H]-	CerP(d18:0/16:0)	Sphingolipids	1.13	-0.18	1.19	618.47	34.85
11	ESI+	[M+H]+	Cer(d18:0/18:0)	Sphingolipids	1.73	-0.05	1.50	568.56	33.93
12	ESI-	[M-H-H2O]-	Ganoderic acid H	Prenol lipids	1.03	-0.14	1.06	553.28	34.79
13	ESI+	[M+H]+	Ceramide (d18:1/16:0)	Sphingolipids	2.3	-0.10	1.42	538.52	35.30
14	ESI-	[M-H]-	Probucol	Carboxylic acids and derivatives	1.59	-0.16	1.02	515.30	51.25
15	ESI-	[M-H]-	Glycocholic acid	Steroids and steroid derivative	1.37	-0.34	1.74	464.30	252.88
16	ESI-	[M-H]-	alpha-Tocopherol	Prenol lipids	2.09	-0.12	2.07	429.37	33.51
17	ESI+	[M+NH4]+	Cholic acid	Steroids and steroid derivatives	3.12	-0.26	1.17	426.31	217.70
18	ESI+	[M+H-H2O]+	Cholesterol	Steroids and steroid derivatives	6.73	-0.14	2.26	369.35	33.93
19	ESI-	[M-H]-	Anacardic acid	Benzene and substituted derivatives	1.10	-0.26	2.60	347.25	79.03
20	ESI+	[M+Na]+	Acetohexamide	Organooxygen compounds	1.04	-0.47	2.24	347.09	253.00
21	ESI-	(M-H2O-H)-	Prostaglandin A1	Fatty Acyls	1.40	-0.22	2.52	317.21	60.16
22	ESI+	[M+H]+	gamma-Glutamylglutamic acid	Carboxylic acids and derivatives	4.69	1.13	3.46	277.09	258.97
23	ESI-	[M-H]-	Sorbitol 6-phosphate	Organooxygen compounds	1.12	0.09	1.20	261.03	295.80

Supplementary Table S4 Differential serum metabolites identified of oat β-glucan (BG) group vs. control (CT) group in the positive and negative mode.

No.	Ions mode	Adduct	Metabolite	HMDB classification	VIP	Log2 (FC)	-Log10(*p*)	m/z	rt(s)
24	ESI+	(M+CH3COO+2H)+	D-Pinitol	Organooxygen compounds	1.11	0.59	3.98	255.11	258.28
25	ESI+	[M+H]+	Anserine	Peptidomimetics	3.56	0.26	1.42	241.13	415.50
26	ESI-	[M-H-C4H8O4]-	Isomaltose	Organooxygen compounds	1.76	0.43	1.15	221.07	98.21
27	ESI-	M-	m-Chlorohippuric acid	Benzene and substituted derivatives	1.25	-0.12	1.10	213.01	165.84
28	ESI+	[M+H]+	Kynurenic acid	Quinolines and derivatives	2.83	0.49	3.52	190.05	190.76
29	ESI-	(M-H)-	Sulfanilamide	Benzene and substituted derivatives	1.08	0.13	1.92	171.03	340.43
30	ESI+	[M+H]+	L-Phenylalanine	Carboxylic acids and derivatives	2.32	0.09	1.09	166.08	256.35
31	ESI+	[M+H-C7H14]+	2-heptyl-4-hydroxyquinoline n-oxide	Quinolines and derivatives	3.16	-0.18	1.07	162.05	79.72
32	ESI-	[M-H-H2O]-	D-psicose	Organooxygen compounds	1.63	0.81	2.47	161.04	97.90
33	ESI+	[M+H-CH3ON]+	L-homoarginine	Carboxylic acids and derivatives	9.62	0.16	1.33	144.10	302.66
34	ESI+	[M+H]+	Stachydrine	Carboxylic acids and derivatives	36.27	0.14	1.82	144.10	270.15
35	ESI-	[M-H]-	Quinolin-2-ol	Quinolines and derivatives	2.50	0.26	2.67	144.04	192.52
36	ESI-	[M-H]-	Malate	Hydroxy acids and derivatives	2.47	0.31	1.43	133.01	412.04
37	ESI-	[M-H-CO2]-	Cis-aconitate	Carboxylic acids and derivatives	1.13	0.11	1.78	129.02	431.62
38	ESI+	[M+H]+	Imidazoleacetic acid	Azoles	1.79	-0.32	1.56	127.05	332.74
39	ESI-	[M-H]-	Methylmalonic acid	Carboxylic acids and derivatives	1.70	0.22	1.24	117.02	384.22
40	ESI+	[M+H-C3H5ClS]+	Sulfallate	Dithiocarbamic acids and derivatives	1.10	0.13	1.12	116.05	34.13
41	ESI-	[M-H]-	Dl-lactate	Hydroxy acids and derivatives	17.95	0.20	1.16	89.025	219.38
42	ESI-	[M-H]-	Pyruvate	Keto acids and derivatives	4.82	0.16	1.02	87.01	124.93

ESI+ was positive ion model, ESI- was negative ion model. FC, fold change, Log2(FC) indicated the logarithmic function of fold change based on 2, Log2 (FC) > 0 indicated the metabolite concentration in oat β-glucan (BG) group were higher than that in control(CT) group, Log2 (FC) < 0 indicated the metabolite concentration in oat β-glucan (BG) group were lower than that in control(CT) group ; Log10(*p*) indicated the logarithmic function of *p*-value based on 10. -Log10(0.1) =1; -Log10(0.05) = 1.3; -Log10(0.01) = 2; -Log10(0.001) = 3. m/z =mass-to-charge ratio; RT = retention time; VIP =variable importance in projection.

3 Supplementary Figures

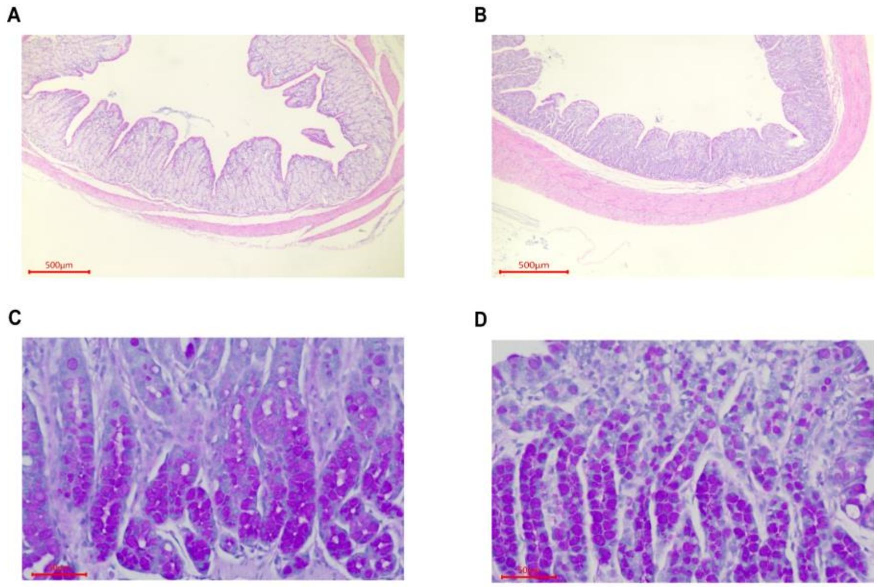

Supplementary Figure S1. (A) and **(B)** were representative images of hematoxylin and eosin-stained colonic sections in control (CT) and oat β-glucan (BG) groups, respectively. Scale bar = 500 μm. **(C)** and **(D)** were representative images of periodic acid–Schiff-stained colonic sections in control (CT) and oat β-glucan (BG) groups, respectively. Scale bar = 50 μm.

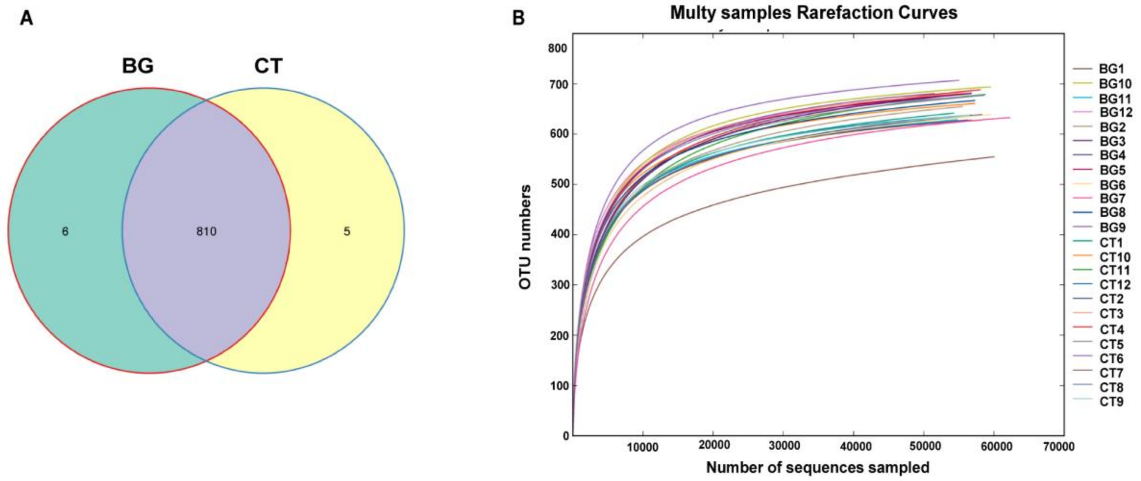

Supplementary Figure S2. (A) The Venn diagram between control (CT) and oat β-glucan (BG) groups. **(B)** The rarefaction curve of control (CT) and oat β-glucan (BG) groups.

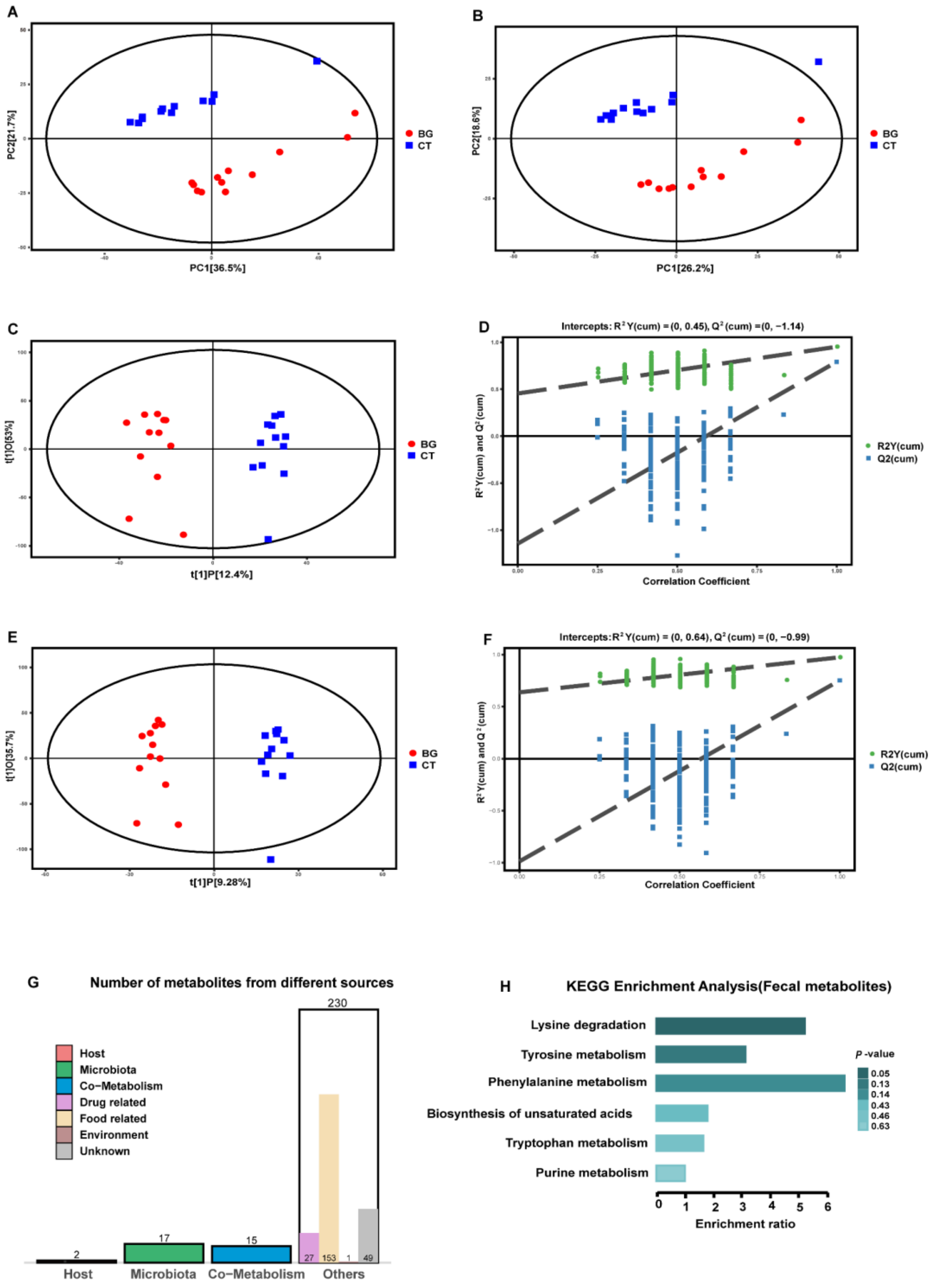

Supplementary Figure S3. (A) and **(B)** Principal component analysis (PCA) score plots for the control (CT) and oat β-glucan (BG) groups colonic content samples analyzed in the positive ion mode and negative ion mode, respectively. **(C)** and **(D)** Orthogonal partial least square discriminant analysis

(OPLS-DA) of scores and permutation test plots for the control (CT) and oat β-glucan (BG) groups colonic content samples analyzed in the positive ion mode, respectively. **(E)** and **(F)** OPLS-DA of scores and permutation test plots for the control (CT) and oat β-glucan (BG) groups colonic content samples analyzed in the negative ion mode, respectively. t[1]P = first principal component, t[1]O = orthogonal principal component. The intercept limit of Q^2, calculated by the regression line, is the plot of Q^2 from the permutation test in the OPLS-DA model. **(G)** Bar plot of the number of colonic metabolites in different categories. **(H)** The KEGG enrichment analysis of colonic metabolites.

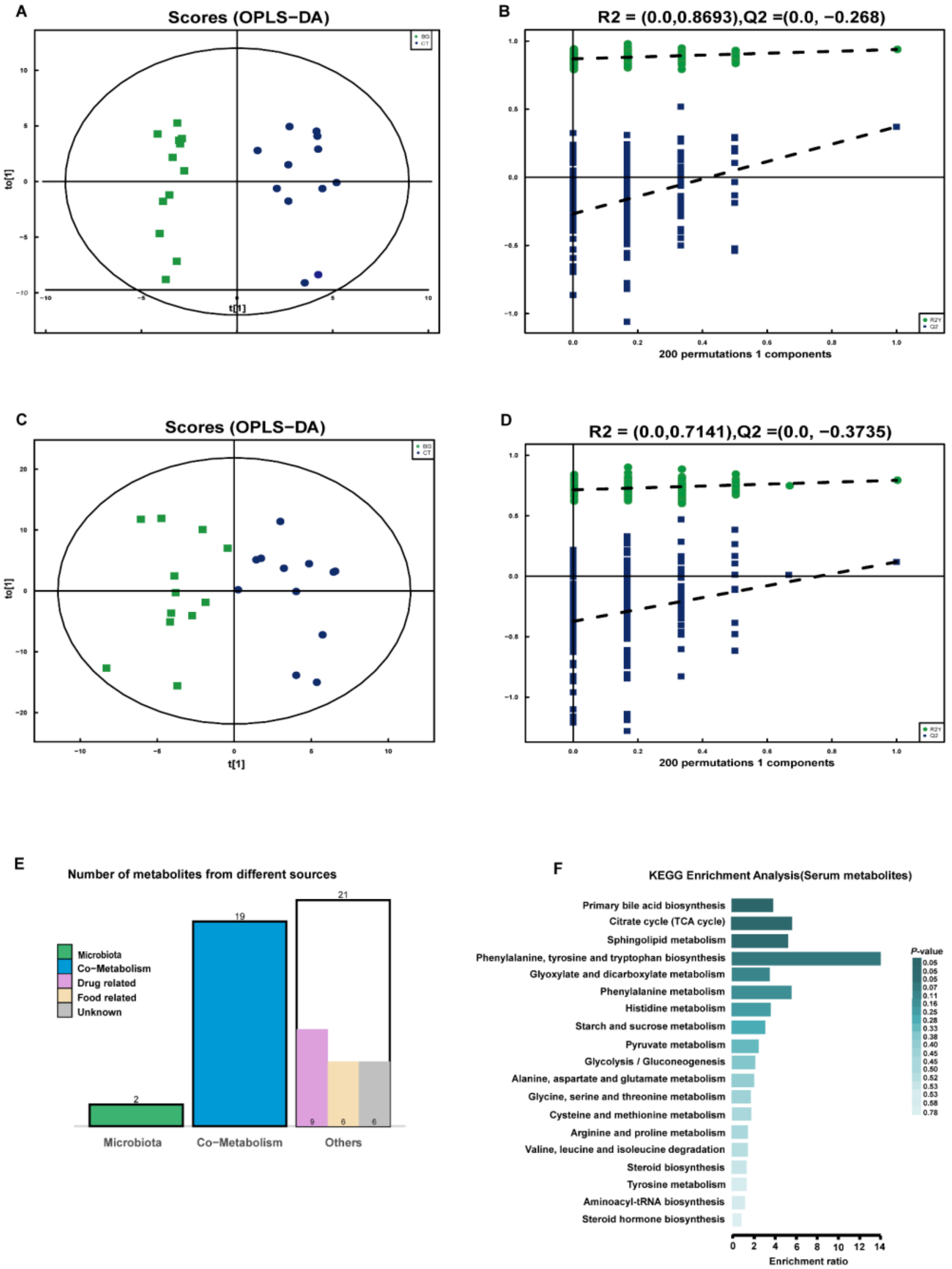

Supplementary Figure S4. (A) and **(B)** OPLS-DA of scores and permutation test plots for the control (CT) and oat β-glucan (BG) groups serum samples analyzed in the positive ion mode, respectively. **(C)**

and **(D)** OPLS-DA of scores and permutation test plots for the control (CT) and oat β-glucan (BG) groups serum samples analyzed in the negative ion mode, respectively. t[1] = first principal component. to[1] = second orthogonal component. The intercept limit of Q2, calculated by the regression line, is the plot of Q^2 from the permutation test in the OPLS-DA model. **(E)** Bar plot of the number of serum metabolites in different categories. **(F)** The KEGG enrichment analysis of serum metabolites.

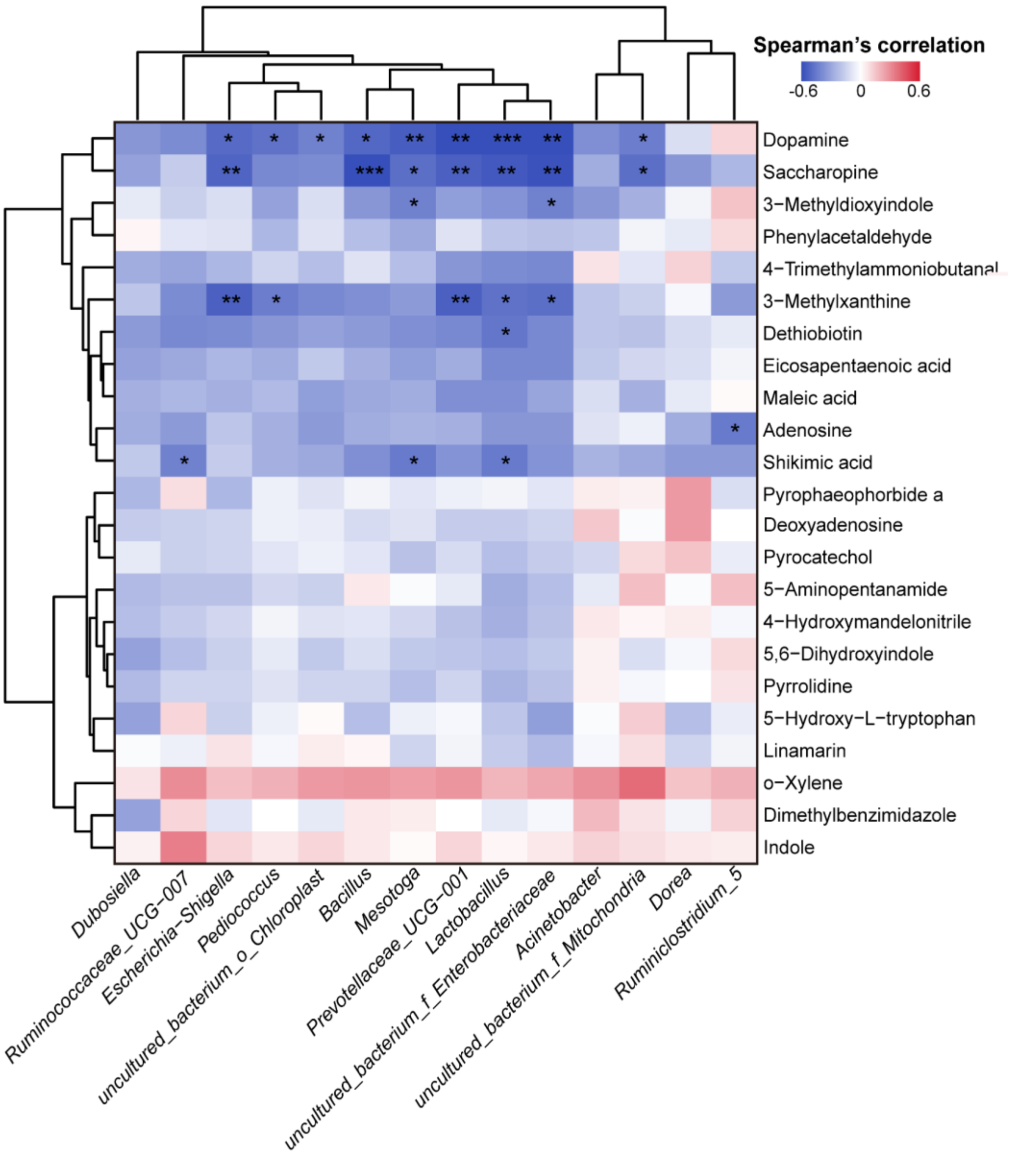

Supplementary Figure S5. Spearman's correlation analysis of the relative abundance of differential bacteria at the genus level and differential intestinal metabolites. * $p < 0.05$ or ** $p < 0.01$ or *** $p < 0.001$. The spearman's correlation coefficient of significant correlations > 0.4 (or < -0.4).

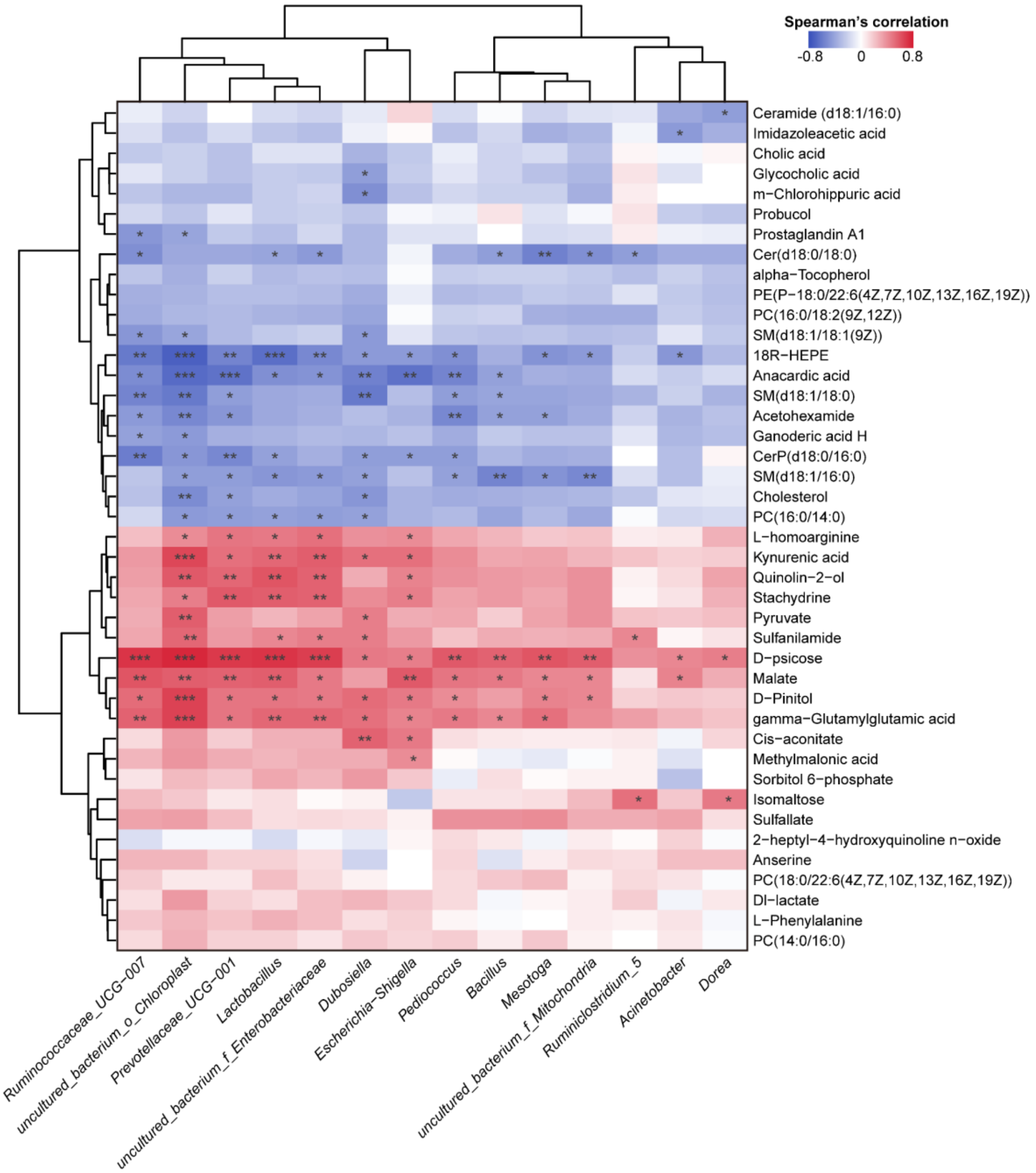

Supplementary Figure S6. Spearman's correlation analysis of the relative abundance of differential bacteria at the genus level and differential serum metabolites. * $p < 0.05$ or ** $p < 0.01$ or *** $p < 0.001$. The spearman's correlation coefficient of significant correlations > 0.4 (or < -0.4).

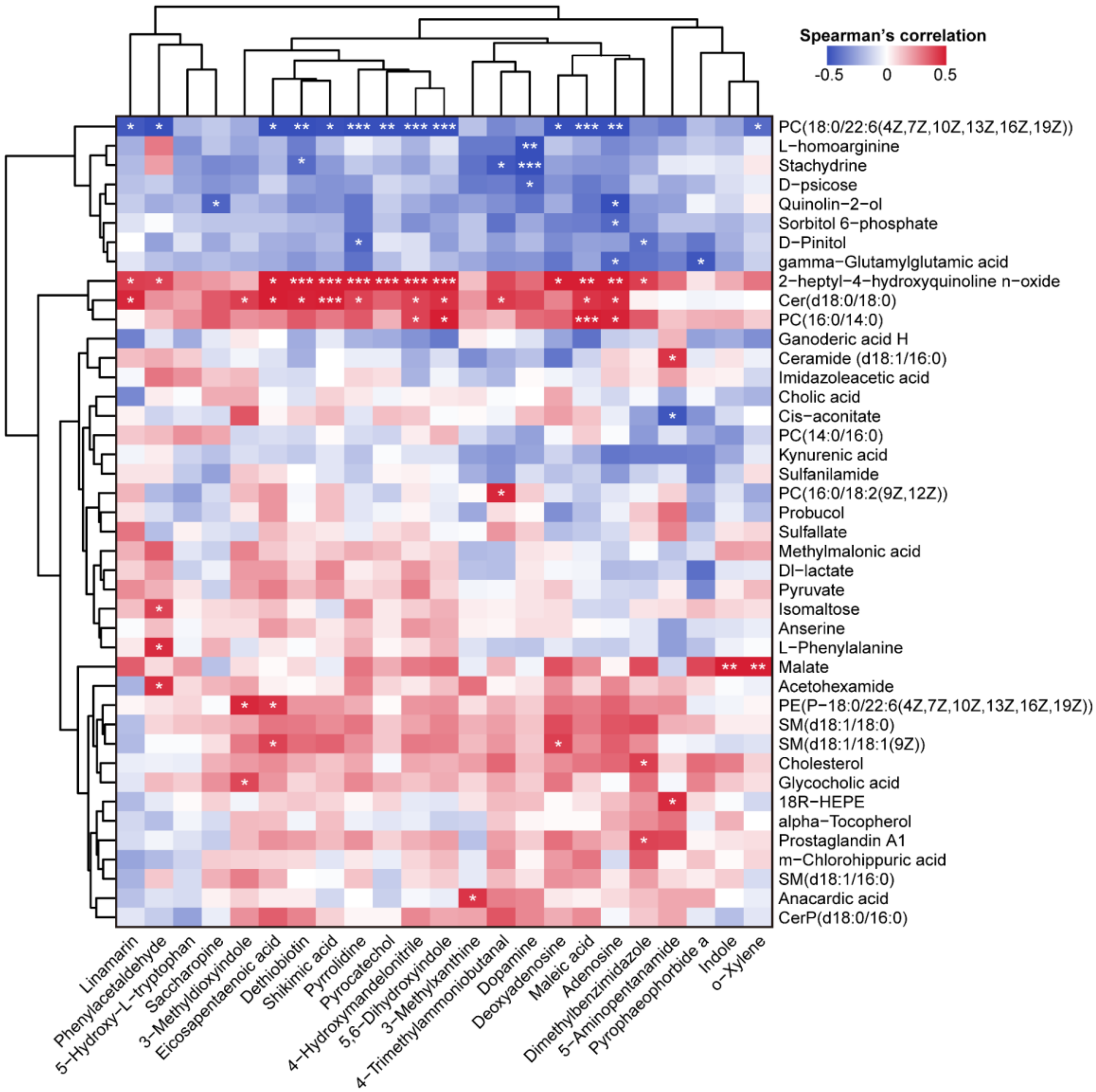

Supplementary Figure S7. Spearman's correlation analysis of differential intestinal metabolites and differential serum metabolites. * $p < 0.05$ or ** $p < 0.01$ or *** $p < 0.001$. The spearman's correlation coefficient of significant correlations > 0.4 (or < -0.4).

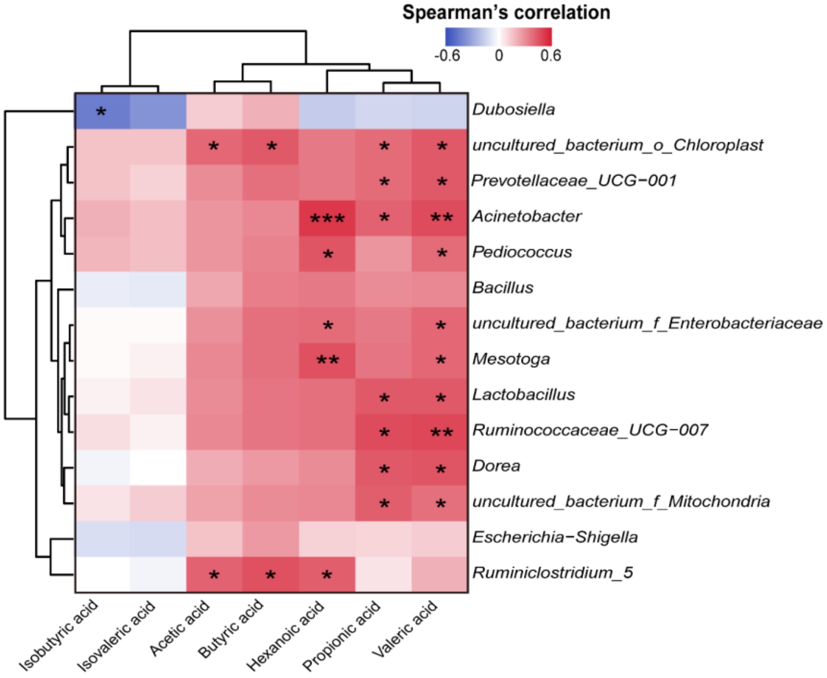

Supplementary Figure S8. Spearman's correlation analysis of the relative abundance of differential bacteria at the genus level and intestinal SCFAs. * $p < 0.05$ or ** $p < 0.01$ or *** $p < 0.001$. The spearman's correlation coefficient of significant correlations > 0.4 (or < -0.4).
